# Understanding Differences in the Body Burden–Age Relationships of Bioaccumulating Contaminants Based on Population Cross Sections versus Individuals

**DOI:** 10.1289/ehp.1104236

**Published:** 2012-01-24

**Authors:** Cristina L. Quinn, Frank Wania

**Affiliations:** Department of Chemistry, and Department of Physical and Environmental Sciences, University of Toronto Scarborough, Toronto, Ontario, Canada

**Keywords:** biomonitoring, cross-sectional trends, environmental modeling, human bioaccumulation, longitudinal trends, time-variant exposure

## Abstract

Background: Body burdens of persistent bioaccumulative contaminants estimated from the cross-sectional biomonitoring of human populations are often plotted against age. Such relationships have previously been assumed to reflect the role of age in bioaccumulation.

Objectives: We used a mechanistic modeling approach to reproduce concentration-versus-age relationships and investigate factors that influence them.

Method: CoZMoMAN is an environmental fate and human food chain bioaccumulation model that estimates time trends in human body burdens in response to time-variant environmental emissions. Trends of polychlorinated biphenyl (PCB) congener 153 concentrations versus age for population cross sections were estimated using simulated longitudinal data for individual women born at different times. The model was also used to probe the influence of partitioning and degradation properties, length of emissions, and model assumptions regarding lipid content and liver metabolism on concentration–age trends of bioaccumulative and persistent contaminants.

Results: Body burden–age relationships for population cross sections and individuals over time are not equivalent. The time lapse between the peak in emissions and sample collection for biomonitoring is the most influential factor controlling the shape of concentration–age trends for chemicals with human metabolic half-lives longer than 1 year. Differences in observed concentration–age trends for PCBs and polybrominated diphenyl ethers are consistent with differences in emission time trends and human metabolic half-lives.

Conclusions: Bioaccumulation does not monotonically increase with age. Our model suggests that the main predictors of cross-sectional body burden trends with age are the amount of time elapsed after peak emissions and the human metabolic and environmental degradation rates.

Biomonitoring studies involve the measurement of chemical concentrations in blood, urine, or tissues of a large number of individuals to assess the exposure of a population to environmental contaminants. Such studies may provide information about the chemicals a population is exposed to, changes in exposure over time, and factors that influence exposure. Numerous cross-sectional biomonitoring studies (Becker et al. 2003; Jursa et al. 2006; Uemura et al. 2008) have reported human body burdens of polychlorinated biphenyls (PCBs) that increase monotonically with age (Figure 1A–C), leading some authors to conclude that bioaccumulation increases with age because of longer exposure (Fernandez et al. 2008; Hardell et al. 2010; James et al. 2002; Naert et al. 2006) or age-dependent changes in metabolism (Ahlborg et al. 1995; Fangstrom et al. 2005). An alternative hypothesis is that older individuals lived during periods of greater PCB exposure (Ahlborg et al. 1995; Patterson et al. 2008; Porta et al. 2008). A recent review by Porta et al. (2008) highlighted that the interplay between age effects and birth cohort effects has yet to be quantitatively evaluated.

**Figure 1:**
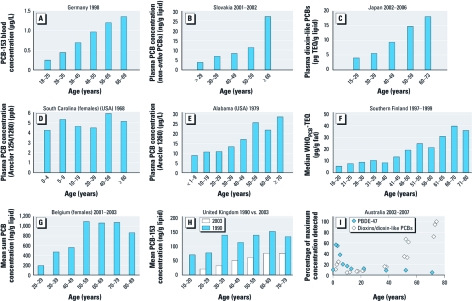
Literature-reported CBATs for PCBs for Germany (*A*; Becker et al. 2003), Slovakia (*B*; Jursa et al. 2006), Japan (*C*; Uemura et al. 2008), the U.S. states of South Carolina (*D*; Finklea et al. 1972) and Alabama (*E*; Kreiss et al. 1981), southern Finland (*F*; Kiviranta et al. 2005), Belgium (*G*; Naert et al. 2006), and the United Kingdom (*H*; Duarte-Davidson et al. 1994; Thomas et al. 2006) and for measurements for both PCB and PBDE for Australia (*I*; Mueller and Toms 2010). Abbreviations: TEQ, toxic equivalent concentration; WHO, World Health Organization.

Not all cross-sectional body burden-versus-age trends (CBATs) for PCBs consistently increase with age however (Figure 1D–H) (Duarte-Davidson et al. 1994; Finklea et al. 1972; Kiviranta et al. 2005; Kreiss et al. 1981; Naert et al. 2006; Thomas et al. 2006). Furthermore, in contrast with data for PCBs, cross-sectional data from Australia for 2002–2007 suggest a decreasing trend in serum concentrations of polybrominated diphenyl ethers (PBDEs) with age (Figure 1I) (Mueller and Toms 2010). Covaci et al. (2008) proposed that opposing age trends for PCBs and PBDEs may suggest that PCBs have reached steady-state levels in human tissues. They also suggest that different exposure routes and metabolic rates could explain this difference. Others have proposed that the body burdens of infants are greater than those of adults because under steady-state conditions, exposure from breast milk is greater than that from adult food sources (Johnson-Restrepo and Kannan 2009).

The mechanistic bioaccumulation model CoZMoMAN (Breivik et al. 2010) suggests that in individuals with constant exposure, lipid-normalized body burdens of persistent PCB congeners may actually decrease after age 50 (Quinn et al. 2011). We propose that the increase in PCB body burden with age observed in cross-sectional population studies is a result of the timing of sample collection relative to peak PCB emissions, which determines the peak in exposure. In the present study, we used the CoZMoMAN model to investigate factors that influence the shape of CBATs and relate these findings to observed CBATs reported in the literature. Ritter et al. (2011) recently applied a pharmacokinetic model to cross-sectional biomonitoring data to estimate intrinsic elimination half-lives of PCBs in humans. They also derived CBATs from their model, which relies on contaminant measurements in food to empirically estimate time trends in exposure. In the present study, we go beyond this approach by applying a mechanistic multimedia fate model to quantify time-variant exposure. Furthermore, our analyses include both pre- and postban populations rather than focusing only on the postban situation.

## Methods

The previously evaluated mechanistic time-variant multimedia model CoZMoMAN (Breivik et al. 2010) was used to estimate CBATs for several chemicals varying in partitioning and degradation properties. CoZMoMAN calculates the transfer of time-variant emissions of an organic chemical through atmospheric, aqueous, and terrestrial compartments and its subsequent bioaccumulation in the food web and transfer to humans through the uptake of contaminated food, air, and water. Each environmental phase is modeled as one compartment, and the human is modeled as two compartments—the digestive tract and the main body (Czub and McLachlan 2004). Internal kinetic distributions are not considered. The model requires information on the contaminant’s octanol–water and octanol–air partition coefficients (log *K*_OW_ and log *K*_OA_, respectively), its environmental and metabolic half-lives, and the historical time trend of its emissions. A detailed discussion of the model can be found in Breivik et al. (2010) and references therein.

**Figure 2:**
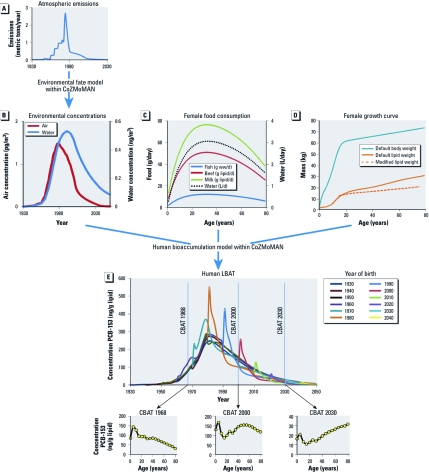
Schematic overview of how the CoZMoMAN model estimates CBATs for PCB-153: historical atmospheric emission estimates (*A*; adapted from Breivik et al. 2010) are used to derive time trends in the concentrations in air and water (*B*) using a physical environmental fate model. These concentrations in exposure-relevant environmental media, as well as assumptions concerning dietary composition (*C*) and growth curves for these women (*D*; adapted from Czub and McLachlan 2004), are used to calculate LBATs for women born every 5 years (*E*) using a human food chain bioaccumulation model. Finally, CBATs for 1968, 2000, and 2030 are obtained by “sampling” the women whose LBATs had been estimated. ww, wet weight.

Cross-sectional data generated through biomonitoring studies are based on groups of different individuals sampled at the same time, whereas the longitudinal estimates derived by CoZMoMAN are for single individuals over their entire lifetimes. For the present analysis, cross-sectional trends were determined from model-derived longitudinal estimates of lipid-normalized concentrations for individual women born at 5-year intervals starting several decades before emissions of the chemical to the environment began and ending several decades after emissions ceased. Each simulated woman was an only child born to a 30-year-old mother and breast-fed for 6 months, each had one child of her own at age 30 that she breast-fed for 6 months, and each died at age 80. The process of deriving CBATs from the longitudinal body burden age trends (LBATs) simulated for individual women is illustrated in Figure 2 for PCB congener 153 (PCB-153) (log *K*_OW_ = 7; log *K*_OA_ = 9.5) assuming the historical atmospheric emissions profile (Figure 2A) (Breivik et al. 2010), dietary intakes for southern Sweden (Figure 2C) (Quinn et al. 2011), changes in body weight and lipid weight with age (Figure 2D) (Czub and McLachlan 2004), and a constant age-independent metabolic rate. Unless otherwise noted, all PCB-153 emissions are assumed to have been released into the atmosphere.

In this example, 17 different LBATs (for 17 individual women 0, 5, 10 . . . 80 years of age) were used to estimate each CBAT, with each LBAT contributing one data point to a given CBAT. For example, to derive a CBAT for PCB-153 measured in a cross-sectional population in the year 2000, the body burden for a 30-year-old is taken from the LBAT estimated for a woman born in 1970, and the body burden of an 80-year-old is taken from the LBAT estimated for a woman born in 1920 (Figure 2E).

In all simulations, we estimated dietary intakes assuming a typical Swedish diet, which corresponds to daily consumption of 50 g dairy lipid, 12 g beef lipid, and 75 g wet weight fish for 25-year-old women (Czub and McLachlan 2004). We assumed that the dietary proportions of dairy, beef, and fish were constant over time but that daily consumption varied with age (Figure 2C) (Moser and McLachlan 2002).

Because the focus of the analyses was on the shape of the CBATs and not on absolute human body burdens, estimated body burdens for individual women (at different ages) were normalized to the average value of the population cross section at that point in time. For each scenario, *C*_N(_*_t_*_)_, the normalized concentration for a woman of age *t*, was calculated as *C_t_*/*C*_avg_, where *C_t_* is the estimated concentration for a woman of age *t* and *C*_avg_ is the average estimated concentration of the population cross section for the given scenario. In other words, the CBATs reflect the body burden of a woman at a given age relative to the average of the entire population.

*Sensitivity analyses.* To identify and better understand the factors that may influence the shape of CBATs, we performed additional hypothetical simulations in a series of sensitivity analyses. The influence of human metabolic half-lives on CBAT shape was evaluated for a hypothetical chemical of log *K*_OW_ 7 and log *K*_OA_ 9.5 (corresponding to the partitioning properties of PCB-153) and five different half-lives of 1, 3, 5, 15, and 50 years.

**Figure 3:**
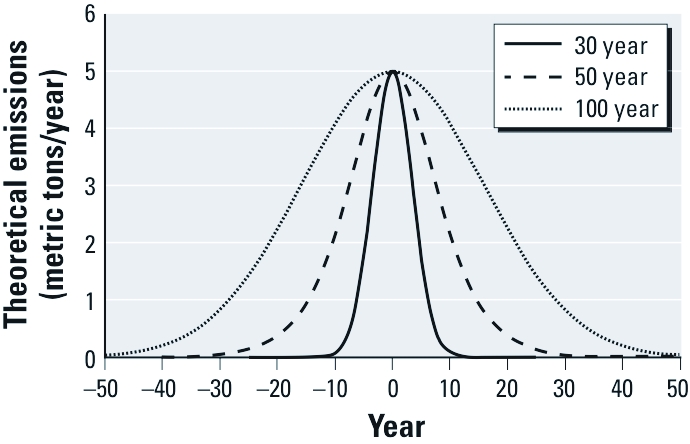
Shape of three theoretical emission scenarios lasting 30, 50, and 100 years.

To explore the influence of the length of the emission period on CBAT shapes, we performed additional simulations for a chemical with the partitioning properties of PCB-153 (log *K*_OW_ = 7; log = *K*_OA_ 9.5) according to three different metabolic half-lives (1, 15, and 50 years) and three hypothetical emission scenarios represented by bell-shaped functions that increased and decreased over 30, 50, and 100 years, respectively (Figure 3). The rate of decline represents emission half-lives of 1.8, 8.5, and 10 years, respectively.

To explore the influence of partitioning properties, we estimated CBATs for four hypothetical partitioning property combinations representing the range of potentially bioaccumulative chemicals in the human food chain (Kelly et al. 2007): log *K*_OW_ 4 and log *K*_OA_ 7.4, log *K*_OW_ 5.6 and log *K*_OA_ 7.2, log *K*_OW_ 7 and log *K*_OA_ 9.7, and log *K*_OW_ 8.7 and log *K*_OA_ 15.3, corresponding to the partitioning properties of α-hexachlorocyclohexane, hexachlorobenzene, *p*,*p*´-dichlorodiphenyldichloroethylene, and decabrominated diphenyl ether, respectively. For these analyses, we assumed a human metabolic half-life of 15 years and the 50-year emission scenario.

We evaluated the influence of model assumptions regarding age trends in lipid content and liver metabolic capacity assuming the partitioning properties of PCB-153 and the 50-year emission scenario. Specifically, we assumed a slower increase in body fat between ages 20 and 80 such that at age 80 the female body lipid content was only 20 kg (27% of body weight) rather than 30 kg (40% of body weight; Figure 2D). Because the body burdens are lipid normalized, a lower body fat content would translate into greater body burdens. To determine if age-dependent metabolic degradation capacity as a function of liver volume could influence CBAT shape, we modified the metabolic degradation rate to be dependent on liver volume according to Alcock et al. (2000), assuming age-dependent changes in liver volume according to van der Molen et al. (1996).

## Results and Discussion

**Figure 4:**
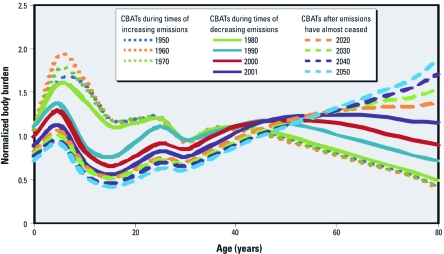
Comparison of CoZMoMAN-generated CBATs for PCB-153 for cross-sectional biomonitoring studies conducted every 10 years between 1950 and 2050, assuming the emission scenario described in Figure 2A.

PCB-153 CBATs calculated every 10 years from 1950 to 2050 for women in Sweden, derived as described above, are shown in Figure 4. The shape of CBATs for PCB-153 depends strongly on the year of sampling relative to the emissions profile, which peaked in 1974 in this scenario. These results highlight the gradual shifts among age groups over time and the influence of the time of sampling relative to the emission time trend, which is crucial to understanding the relation between age and PCB body burden based on cross-sectional data collected at different points in time.

A pattern common to all of the CBATs is a drop in estimated PCB-153 levels in women at age 30, when all of the women included in the simulations give birth to a child (Quinn et al. 2011). For an entirely nulliparous female population, the CBAT minimum would actually occur at age 15 [see Supplemental Material, Figure 1 (http://dx.doi.org/10.1289/ehp.1104236)]. In a realistic population, the effects of reproduction on PCB-153 concentrations in a cross-sectional sample would be obscured because of variation in the age of childbirth, the number of children, and breast-feeding practices.

*CBATs during times of increasing emissions.* When emissions are still increasing (i.e., 1950–1970), CoZMoMAN predicts the highest body burdens in females around 5 years of age (Figure 4). This reflects the combined effects of increasing prenatal exposure with each generation and the relatively low body lipid content at age 5 (Figure 2D). In other words, 5-year-olds in a biomonitoring study conducted when emissions are increasing will have a greater body burden than will 15-year-olds because they were born into a more contaminated environment and because they have yet to go through a substantial growth dilution phase during their teens. The model predicts that PCB levels will decrease in women > 40 years of age sampled during times of increasing emissions, not because levels decrease in individuals with age but because women who were > 40 years of age when these cross-sectional samples were assembled were not exposed until later in life.

*CBATs during times of decreasing emissions.* The age at which the maximum body burden occurs in a CBAT during times of decreasing emissions depends upon the amount of time elapsed since the emissions peaked. That is, the age at which the maximum body burden occurs in adults will increase as the time between the peak of emissions and cross-sectional sampling increases (Figure 4). In CBATs representing the period after emissions have completely ended, there is a rapid increase in body burden with age because the contaminated population is older and exposure is relatively low in younger members of the population. The Michigan fish-eater cohort, which is one of the few longitudinal data sets reported in the literature (Tee et al. 2003), illustrates this effect. In 1980, serum PCB concentrations were greatest for individuals 50–59 years of age. Ten years later, serum PCB concentrations were highest for the same group of people, who were now 60–69 years of age. This supports our interpretation that the trend of increasing body burden with age observed in cross-sectional PCB biomonitoring studies is due to an emissions-related cohort effect rather than an age-dependent decline in metabolism (Ahlborg et al. 1995; Fangstrom et al. 2005) or bioaccumulation (Fernandez et al. 2008; Hardell et al. 2010; James et al. 2002; Naert et al. 2006).

*Comparison of measured and predicted CBATs for PCBs.* Our model results suggest that the CBAT reported by Finklea et al. (1972), based on a population from the U.S. state of South Carolina sampled in 1968—which is the only CBAT that did not show a clear relationship between age and PCB body burden (Figure 1D)—is consistent with expectations given that the population was sampled at a time close to the peak in PCB emissions. The CBAT from Finklea et al. (1972) is consistent with the CBAT estimated by CoZMoMAN for 1980. In contrast, almost all other CBATs shown in Figure 1 show increasing body burdens with age, which is consistent with the CoZMoMAN-predicted CBATs for the time periods when these samples were collected, which in most cases were > 30 years after the PCB emission peak in the early 1970s. Biomonitoring studies focusing on adults would not resolve the peak in body burden that CoZMoMAN predicts for children. However, a survey of PCBs in children from the Faroe Islands in 1986–1987 revealed that the serum concentrations of PCBs in 7-year-olds were two to three times greater than those in 14-year-olds and two to eight times greater than those measured in newborn cord blood (Barr et al. 2006). Results of the GerES IV (German Environmental Survey 2003/06) for children indicated that 7- to 8-year-olds had 20% greater PCB serum concentrations than did 12- to 14-year-olds (Becker et al. 2008). These studies support the influence of growth dilution on the relation between age and PCB body burden.

**Figure 5:**
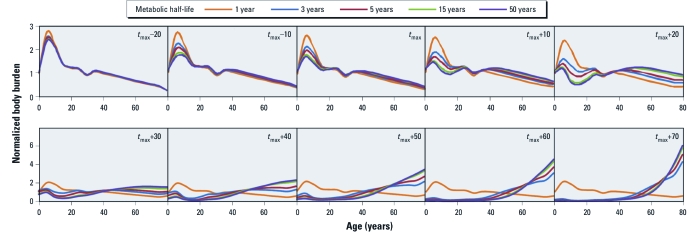
Comparison of CoZMoMAN-generated CBATs for chemicals with log *K*_OW_ 7 and log *K*_OA_ 9.5 and a human metabolic half-life of 1, 3, 5, 15, and 50 years, assuming a bell-shaped emission scenario lasting 50 years. Note that the *y*-axis scale is smaller for the top row than for the bottom row *y*-axis.

*Sensitivity analyses.* Of the parameters evaluated using sensitivity analyses, the metabolic half-life in humans had the greatest influence on CBAT shape (Figure 5). For sampling times before the peak in emissions (*t*_max_), the human metabolic half-life has little influence on CBAT shape. After emission peak (years > *t*_max_), the CBAT shape is similar for all sampling times when the half-life is ≥ 15 years, but for chemicals with half-lives of 3–5 years, the transition between CBAT shapes over time is delayed by 10–20 years relative to substances with a half-life of 15 years. Over the long term, the same CBAT shapes are observed for chemicals with a half-life of 3 years as for chemicals with a half-life of 15 years. However, for a chemical with a half-life of 1 year, the CBAT shape is always the same regardless of sampling time, and only the relative intensity of the maximum changes over time [see Supplemental Material, Figure 2 (http://dx.doi.org/10.1289/ehp.1104236)].

Recent work by Ritter et al. (2011) showed that CBATs could be used to derive information on the intrinsic human elimination half-life. Observations from the present analysis suggest that the method described by Ritter et al. (2011) to derive such half-lives from CBATs will work better late in the emissions cycle (*t* > *t*_max_ + 20 years) and when the half-life is between 1 and 10 years. The CBAT shapes for chemical half-lives outside of this regime will be very similar (Figure 5).

For all emission scenarios, the CBATs remained unchanged with time for a human metabolic half-life of 1 year [Supplemental Material, Figure 3 (http://dx.doi.org/10.1289/ehp.1104236)]. For a 100-year emission scenario, the CBAT shape was also unchanged with time for a 15-year human metabolic half-life but showed time-dependent behavior with a 50-year human metabolic half-life. When the metabolic half-life was 15 and 50 years, the 30- and 50-year emission periods yielded similar CBATs at each time point. From these calculations, we hypothesize that for chemicals with a human metabolic half-life similar to or shorter than the emissions half-life, the CBAT shape will be constant over all time points. For chemicals with human metabolic half-life greater than the emissions half-life, temporal transitions in CBAT shapes will be observed.

Within the investigated range of partitioning properties, virtually no impact on the shape of the CBATs was observed [Supplemental Material, Figure 4 (http://dx.doi.org/10.1289/ehp.1104236)]. This is because the LBAT curves for individuals do not change significantly except for the magnitude of bioaccumulation. This finding is supported by the work of Arnot et al. (2010), who observed similar age patterns in human dietary intake of organic contaminants with a wide range of partitioning properties. Model assumptions regarding lipid content and liver metabolic capacity also had little impact on CBAT shapes (Supplemental Material, Figure 5).

*CBATs of PCB mixtures.* PCB concentrations in human tissues are often reported as the sum of several detected congeners (e.g., dioxin-like PCBs). Each congener will have a unique metabolic half-life, partitioning properties, and possibly also emissions profiles. To determine how the combination of these traits for individual congeners influences the CBATs of PCB mixtures, we calculated a CBAT for the combination of PCB congeners 52, 101, 118, 138, 153, and 180 [see Supplemental Material, Table 1, Figure 6 (http://dx.doi.org/10.1289/ehp.1104236)] for realistic Swedish emissions scenarios (Breivik et al. 2010). From this calculation, it is apparent that the CBAT shapes for the PCB mixture resemble closely the CBATs of the congeners with the longest metabolic half-life (Supplemental Material, Figure 6B), because those congeners contribute the most to the PCB body burden (Supplemental Material, Figure 6A).

*How do the CBATs of PCBs and PBDEs compare?* We propose that the CBAT shapes in any given sampling year should look significantly different for PCBs and PBDEs based solely upon differences in emissions trends over time and human metabolic half-lives. Although the production of PCBs has been banned globally for several decades (Breivik et al. 2007), the production of PBDEs is still increasing in some areas (Ward et al. 2008). Thus, current cross-sectional biomonitoring efforts for PBDEs will yield CBATs with different shapes than CBATs for PCBs. In addition, the apparent elimination half-life of PBDEs is estimated to be several months (Thuresson et al. 2006), compared with elimination half-lives in excess of a decade for highly bioaccumulative PCBs (Brown et al. 1989). CBATs estimated for chemicals with human metabolic half-lives of 1 year and 15 years are therefore relevant to a comparison of CBATs for PBDEs versus PCBs (Figure 5). At *t*_max_ – 20 years, CBATs for both compounds suggest that children at 5 years of age have greater body burdens than do either infants or adult women, consistent with previously reported PBDE biomonitoring studies (Sjodin et al. 2008; Toms et al. 2008, 2009). At *t*_max_ + 20 years, the CBAT for a chemical with a human metabolic half-life of 1 year still presents the same age trends, but the CBAT for a chemical with a 15-year metabolic half-life shows increasing cross-sectional body burdens from 10 to 50 years of age. Because PBDEs are better represented by a metabolic half-life of 1 year than 15 years (Thuresson et al. 2006), we propose that CBATs for PBDEs will exhibit the same CBAT shape regardless of the sampling year. In addition, the shape of CBATs for PCBs and PBDEs would be similar only if they were both based on population cross sections sampled during periods of increasing emissions.

## Conclusions

Our analysis of CBAT shapes and the influence of various model parameters suggests that the most important influences on the shape of cross-sectional time trends are the temporal relationship between the emissions scenario time trend and the biomonitoring sample collection period, and the human metabolic half-life. Although the extent of bioaccumulation of a chemical is also influenced by its partitioning properties, the length of the emissions period, and lipid content and liver clearance rates in the population, the age trend in cross-sectional studies is largely unaffected by these factors. Our results suggest that the observed increase in PCB body burdens for elderly individuals is consistent with an emissions-related cohort effect, and that differences in CBAT shapes for PCBs and PBDEs are consistent with differences in human metabolic rates and emission scenarios.

## Supplemental Material

(532 KB) PDF
