# Supplementary material for: Understanding Differences in the Body Burden–Age Relationships of Bioaccumulating Contaminants Based on Population Cross Sections versus Individuals
Source: Environ Health Perspect. 2012 Jan 24;120(4):554–9. doi: 10.1289/ehp.1104236 (PMC3339463; doi:10.1289/ehp.1104236)
Supplement: (532 KB) PDF [file ehp.1104236.s001.pdf]

Supplementary Material for

Understanding Differences in the Body Burden-Age Relationships of Bioaccumulating Contaminants based on Population Cross-sections versus Individuals

Cristina L. Quinn and Frank Wania[1]

Department of Chemistry and Department of Physical and Environmental Sciences, University of Toronto Scarborough, Toronto, Ontario, Canada M1C 1A4

[1]Corresponding author: Phone: 416-287-7225, Email: frank.wania@utoronto.ca

Table of Contents

**Supplementary Material, Figure 1**	CoZMoMAN-generated cross-sectional body burden age trends for PCB-153 assuming sampling of *nulliparous* Swedish females in contrast to CBATs for women who give birth to a single child at age 30. t_{max} refers to year of maximum atmospheric emissions.	2
**Supplementary Material, Figure 2**	CoZMoMAN-generated cross-sectional body burden age trends for a chemical with a human metabolic half-life of 1 year. t_{max} refers to year of maximum atmospheric emissions.	2
**Supplementary Material, Figure 3**	Comparison of CoZMoMAN-generated CBATs for a chemical with log K_{OW} 7 and log K_{OA} 9.5 and a human metabolic half-life of 1 year, 15 years or 50 years using bell-shaped emission scenarios lasting 30, 50, and 100 years. t_{max} refers to year of maximum atmospheric emissions.	3-4
**Supplementary Material, Figure 4**	Comparison of CoZMoMAN-generated cross-sectional body burden age trends for chemicals with four different partitioning property combinations and a human metabolic half-life of 15 years using bell-shaped emission scenarios lasting 50 years. t_{max} refers to year of maximum atmospheric emissions.	4
**Supplementary Material, Figure 5**	Comparison of CoZMoMAN-generated cross-sectional body burden age trends making different model assumptions t_{max} refers to year of maximum atmospheric emissions.	5
**Supplementary Material, Figure 6**	CoZMoMAN-generated CBATS for 6 PCB congeners (52, 101, 118, 138, 153 and 180) and their sum using congener-specific partitioning and degradation properties (see Table 1 for details) and realistic emission scenarios for Sweden (Breivik K, Czub G, McLachlan MS, Wania F. 2010. Towards an understanding of the link between environmental emissions and human body burdens of PCBs using CoZMoMAN. Environ Int 36:85-91). **(A)** CBATs are plotted as lipid-normalized body burdens. **(B)** CBATs are plotted as population-normalized body burdens.	6
**Supplementary Material, Table 1**	Summary of model parameters used in the various CoZMoMAN simulations.	7

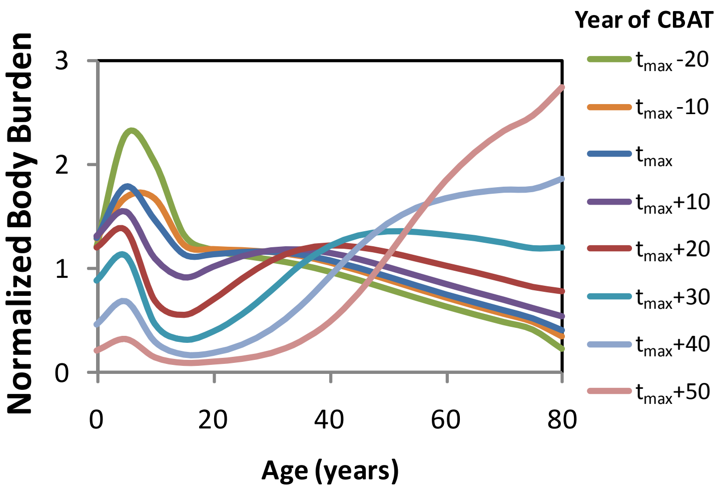

Supplementary Material, Figure 1: CoZMoMAN-generated cross-sectional body burden age trends for PCB-153 assuming sampling of *nulliparous* Swedish females in contrast to CBATs for women who give birth to a single child at age 30. t_{max} refers to year of maximum atmospheric emissions.

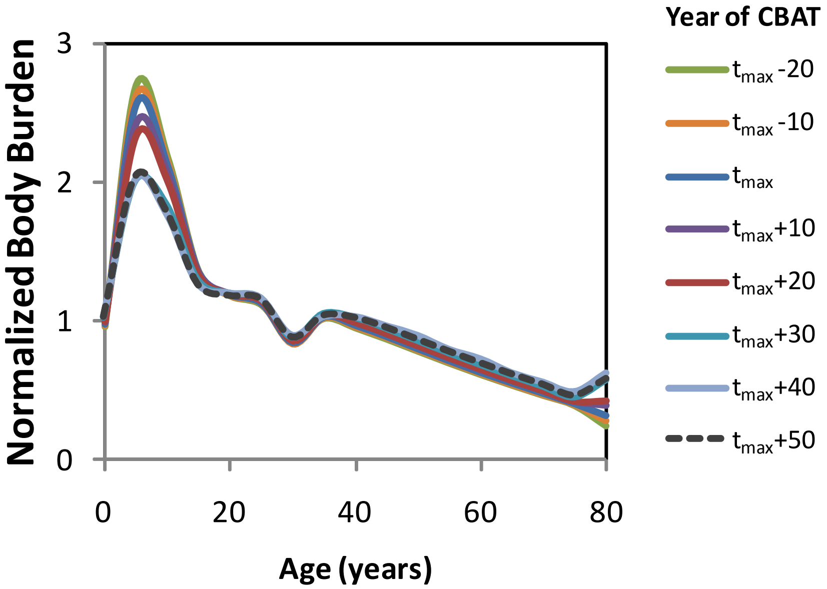

Supplementary Material, Figure 2: CoZMoMAN-generated cross-sectional body burden age trends for a hypothetical chemical with log K_{OW} 7 and log K_{OA} 9.5 emitted to the atmosphere over a 50 period with a human metabolic half-life of 1 year. t_{max} refers to year of maximum atmospheric emissions.

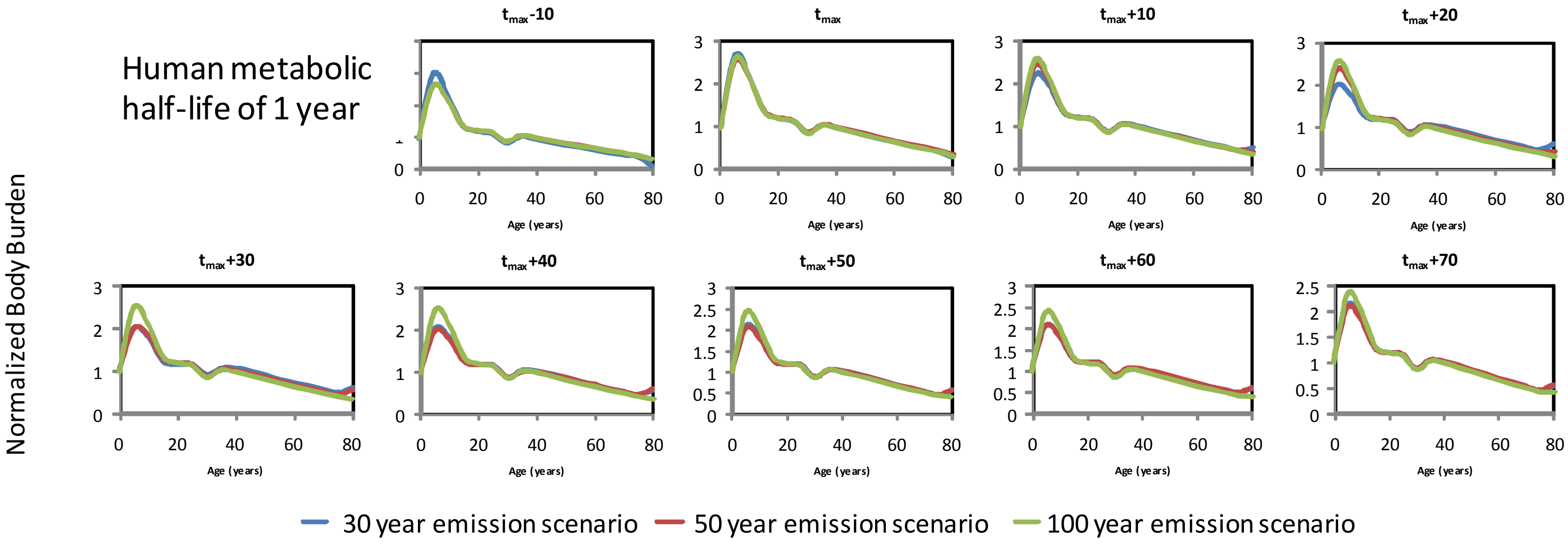
Human metabolic half-life of 1 year
Normalized Body Burden
t_{max}-10
t_{max}
t_{max}+10
t_{max}+20
t_{max}+30
t_{max}+40
t_{max}+50
t_{max}+60
t_{max}+70
Age (years)
30 year emission scenario
50 year emission scenario
100 year emission scenario

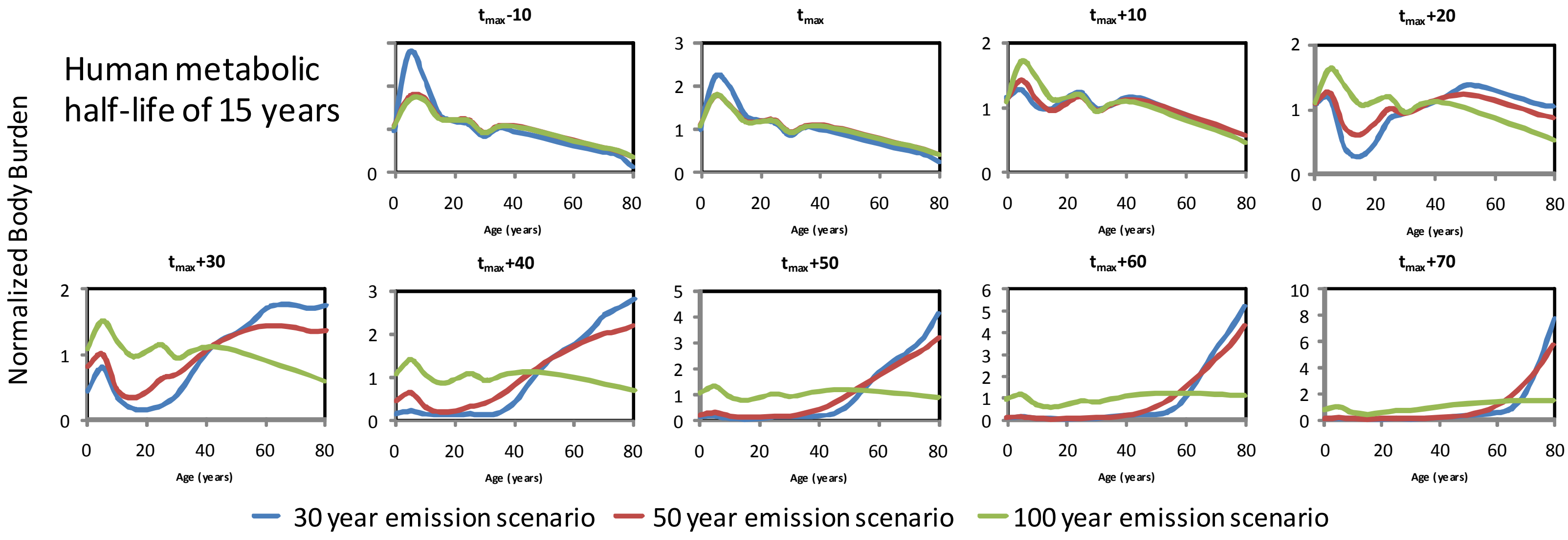
Human metabolic half-life of 15 years
Normalized Body Burden
t_{max}-10
t_{max}
t_{max}+10
t_{max}+20
t_{max}+30
t_{max}+40
t_{max}+50
t_{max}+60
t_{max}+70
Age (years)
30 year emission scenario
50 year emission scenario
100 year emission scenario

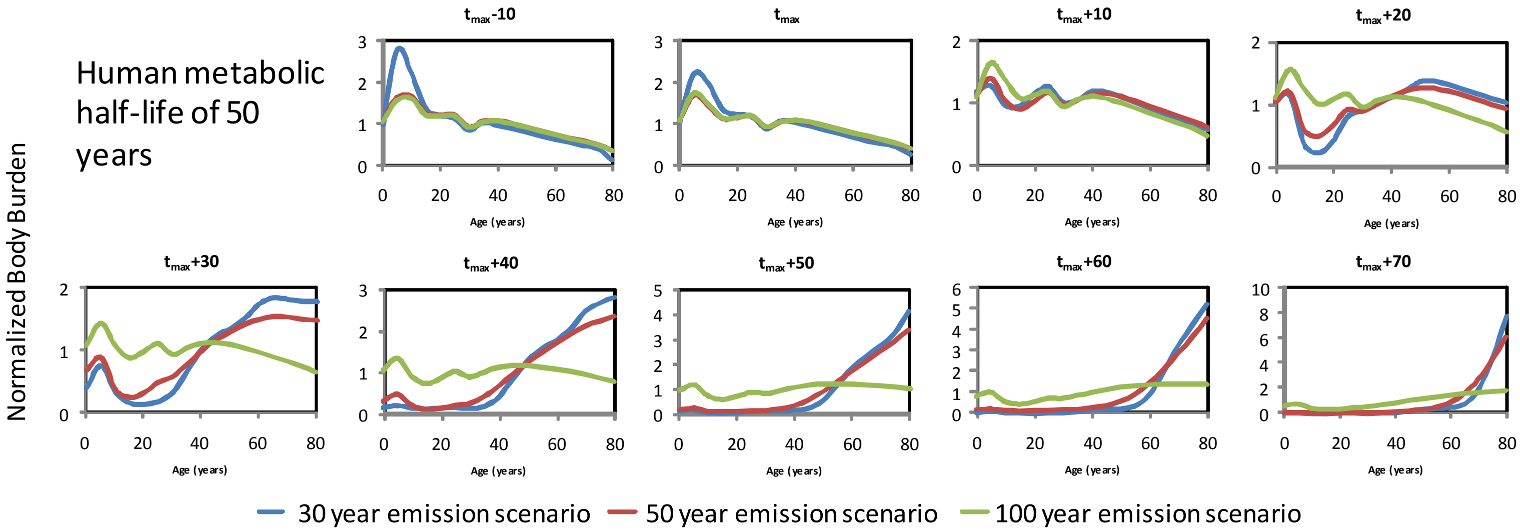

Supplementary Material, Figure 3: Comparison of CoZMoMAN-generated CBATs for a chemical with log K_{OW} 7 and log K_{OA} 9.5 and a human metabolic half-life of 1 year, 15 years or 50 years using bell-shaped emission scenarios lasting 30, 50, and 100 years. t_{max} refers to year of maximum atmospheric emissions.

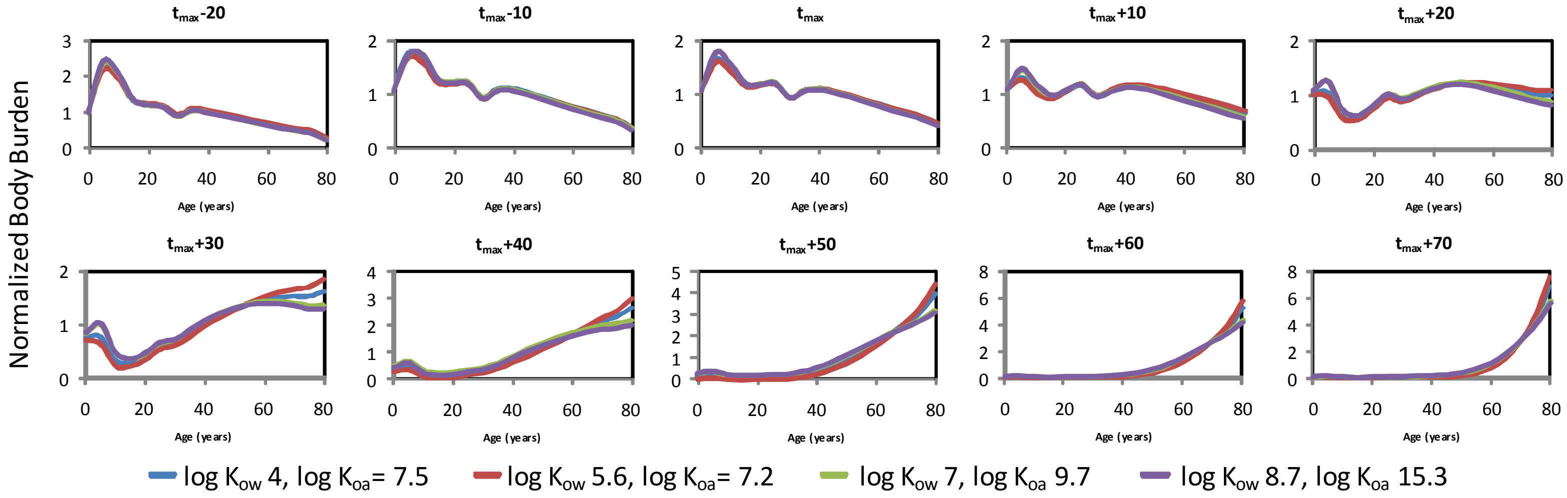

Supplementary Material, Figure 4: Comparison of CoZMoMAN-generated cross-sectional body burden age trends for four chemicals with differing partitioning property combinations and a human metabolic half-life of 15 years using a bell-shaped emission scenario lasting 50 years. t_{max} refers to year of maximum atmospheric emissions.

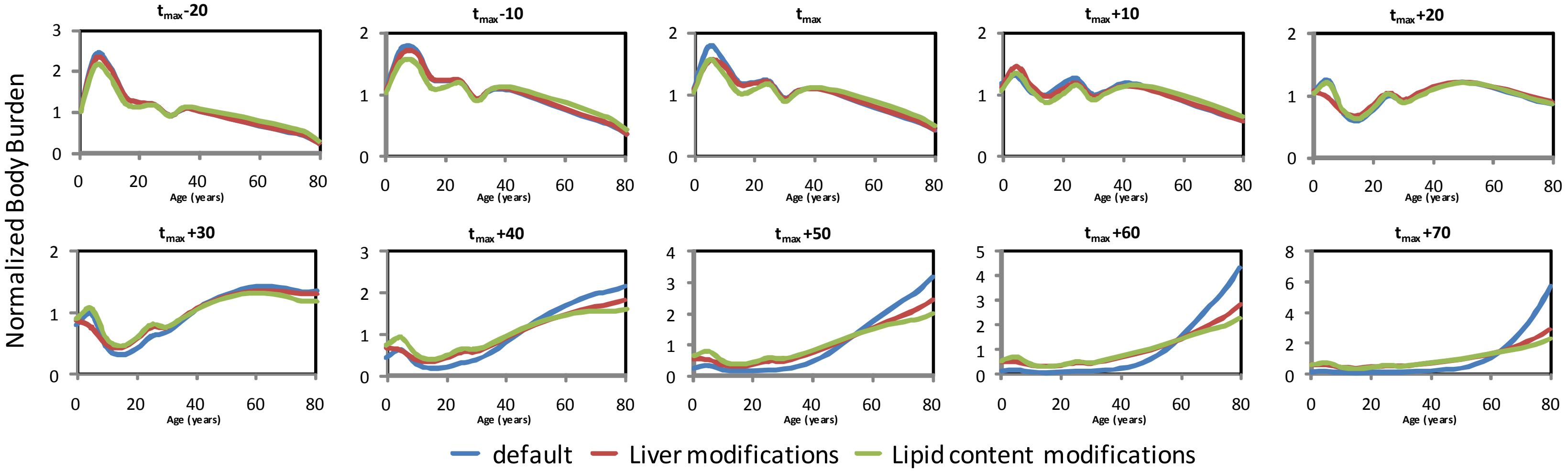

Supplementary Materials, Figure 5: Comparison of CoZMoMAN-generated cross-sectional body burden age trends for a chemical with log K_{OW} 7 and log K_{OA} 9.5 (i.e. those of PCB-153) and a human metabolic half-life of 15 years using a bell-shaped emission scenario lasting 50 years while making different model assumptions concerning the lipid content with age (see Figure 2D) and the metabolizing capacity of the liver with age. t_{max} refers to year of maximum atmospheric emissions.

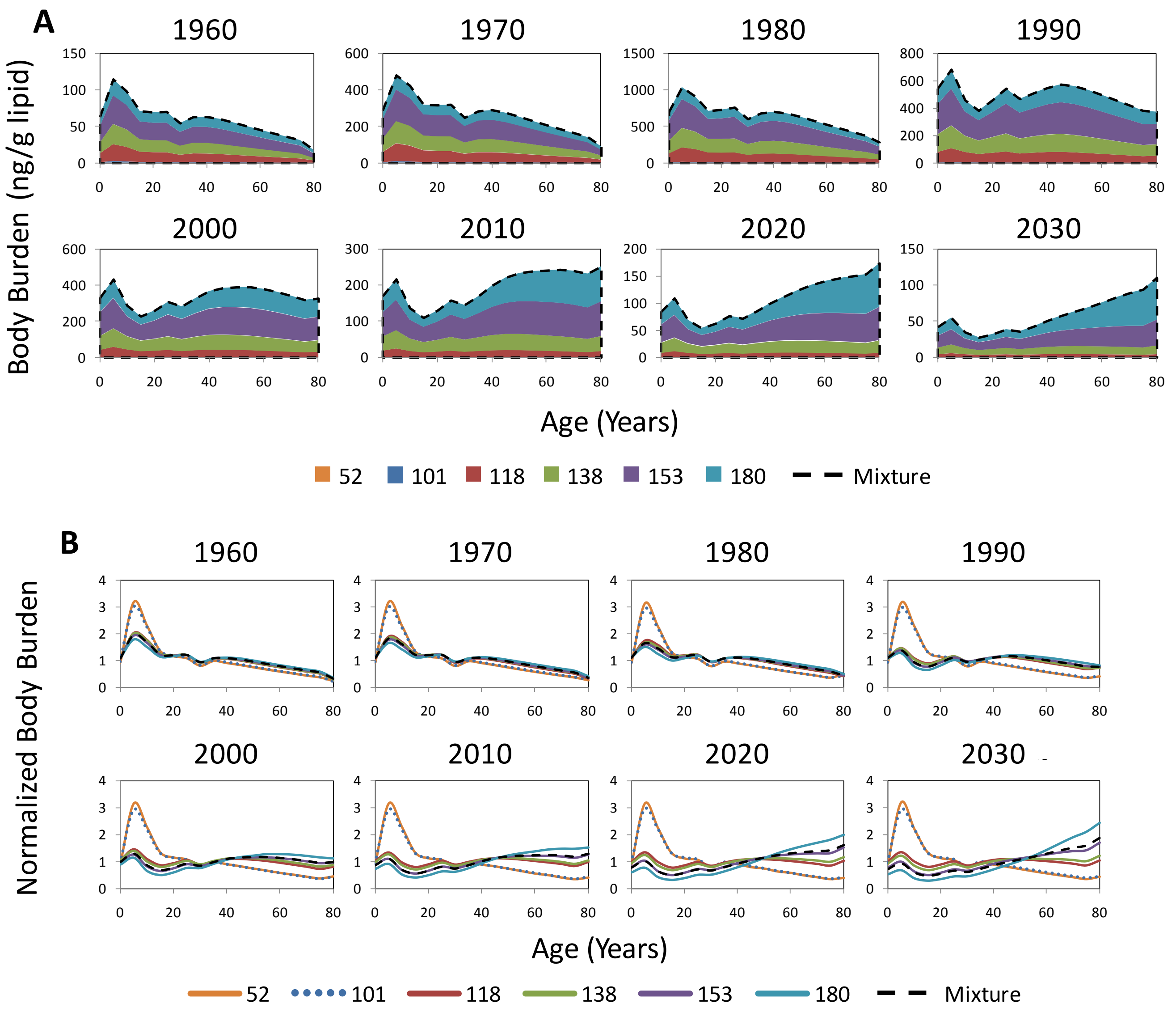

Supplementary Materials, Figure 6: CoZMoMAN-generated CBATS for 6 PCB congeners (52, 101, 118, 138, 153 and 180) and their sum using congener-specific partitioning and degradation properties (see Table 1 for details) and realistic emission scenarios for Sweden (Breivik K, Czub G, McLachlan MS, Wania F. 2010. Towards an understanding of the link between environmental emissions and human body burdens of PCBs using CoZMoMAN. Environ Int 36:85-91). **(A)** CBATs are plotted as lipid-normalized body burdens. **(B)** CBATs are plotted as population-normalized body burdens.

Supplementary Materials, Table 1: Summary of model parameters used in the various CoZMoMAN simulations

	**log K_{OW}**	**log K_{OA}**	**Half-life (years)**	**Emissions Scenario**	**Model assumptions**	**Results**
Realistic conditions for PCB-153	7 (PCB-153) [a]	9.5 (PCB-153) [a]	15	Realistic for Sweden	Model default	Figure 2, Figure 3
					Nulliparous females only	Supplementary Materials, Figure 1
Influence of metabolic half life	7 (PCB-153) [a]	9.5 (PCB-153) [a]	1, 3 ,5, 15, 50	50 years	Model default	Figure 4
CBAT shapes for chemical with metabolic half-life of 1 year	7 (PCB-153) [a]	9.5 (PCB-153) [a]	1	50 years	Model default	Supplementary Materials, Figure 2
Relative size of emission half life and metabolic half life	7 (PCB-153) [a]	9.5 (PCB-153) [a]	1	30, 50, 100 years	Model default	Supplementary Materials, Figure 3
	7 (PCB-153) [a]	9.5 (PCB-153) [a]	15			Supplementary Materials, Figure 3
	7 (PCB-153) [a]	9.5 (PCB-153) [a]	50			Supplementary Materials, Figure 3
Influence of partitioning properties	4 [b]	7.5 [b]	15	50 years	Model default	Supplementary Materials, Figure 4
	5.6 [c]	7.2 [c]	15			
	7 [d]	9.7 [d]	15			
	8.7 [e]	15.3 [e]	15			
Influence of model assumptions	7	9.5	15	50 years	Default, lipid modifications, liver modifications	Supplementary Materials, Figure 5
Realistic conditions for various PCBs	5.9 (PCB-52) [a]	8.2 (PCB-52) [a]	0.1 [f]	Realistic for Sweden	Model default	Supplementary Materials, Figure 6
	6.3 (PCB-101) [a]	8.8 (PCB-101) [a]	0.4 [f]			
	6.7 (PCB-118) [a]	9.4 (PCB-118) [a]	6.3 [f]			
	7.2 (PCB-138) [a]	9.7 (PCB-138) [a]	8.7 [f]			
	7.2 (PCB-180) [a]	10.2 (PCB-180) [a]	231 [f]			

[a] Properties according to Li NQ, Wania F, Lei YD, Daly GL. 2003. A comprehensive and critical compilation, evaluation, and selection of physical-chemical property data for selected polychlorinated biphenyls. J. Phys. Chem. Ref. Data 32:1545-1590. [b] Representative of α-hexachlorocyclohexane (Xiao H, Li NQ, Wania F. 2004. Compilation, evaluation, and selection of physical-chemical property data for α-, β-, and γ-hexachlorocyclohexane. J. Chem. Eng. Data 49:173-185). [c] Representative of hexachlorobenzene (Shen L, Wania F. 2005. Compilation, evaluation, and selection of physical-chemical property data for organochlorine pesticides. J. Chem. Eng. Data 50:742-768), [d] Representative of *p,p'*-dichlorodiphenyl dichloroethylene (Shen L, Wania F. 2005. Compilation, evaluation, and selection of physical-chemical property data for organochlorine pesticides. J. Chem. Eng. Data 50:742-768). [e] Representative of decabrominated diphenyl ether (Wania F, Dugani CB. 2003. Assessing the long-range transport potential of polybrominated diphenyl ethers: A comparison of four multimedia models. Environ. Toxicol. Chem. 22:1252-1261). [f] According to Brown, JF. 1994. Determination of PCB metabolic, excretion, and accumulation rates for use as indicators of biological response and relative risk. Environ Sci Technol. 28:2295-2305.
